# Effectiveness of Time-Restricted Eating with Caloric Restriction vs. Caloric Restriction for Weight Loss and Health: Meta-Analysis

**DOI:** 10.3390/nu15234911

**Published:** 2023-11-24

**Authors:** Tanja Črešnovar, Bernarda Habe, Zala Jenko Pražnikar, Ana Petelin

**Affiliations:** Faculty of Health Sciences, University of Primorska, Polje 42, 6310 Izola, Slovenia; tanja.cresnovar@fvz.upr.si (T.Č.); bernarda.habe@fvz.upr.si (B.H.); zala.praznikar@fvz.upr.si (Z.J.P.)

**Keywords:** time-restricted eating, caloric restriction, overweight, obesity, weight loss, lipid profile, fasting glucose

## Abstract

Time-restricted eating (TRE) is an increasingly popular dietary strategy for weight loss. Recent studies suggest that combining TRE with caloric restriction (CR) may have more favorable effects on both physical and biochemical aspects when compared with CR alone. Therefore, we performed a meta-analysis to compare the effects of TRE with CR vs. CR alone on anthropometric and biochemical measures in overweight or obese adults. We reviewed articles from PubMed, Web of science, EMBASE, and the Cochrane Library published before 25 May 2023. The meta-analysis incorporated data from seven randomized controlled trials of nine interventions, with a total of 231 participants in the TRE with CR group and 227 participants in the CR-only group. Data were analyzed using RewMan version 5.4.1. All results in our meta-analysis were described as mean difference (MD) with 95% confidence interval (Cl). Results showed that TRE with CR compared to CR alone resulted in significantly greater reductions in body weight (MD: −2.11 kg, 95% CI: −2.68 kg to −1.54 kg, *p* = < 0.00001, *I*^2^ = 42%), body fat mass (MD: −0.75 kg, 95% CI: −1.35 kg to −0.16 kg, *p* = 0.01; *I*^2^ = 0%), and waist circumference (MD: −1.27 cm, 95% CI: −2.36 cm to −0.19 cm, *p* = 0.02, *I*^2^ = 0%), while no additional impact of TRE in combination with CR in comparison to CR on serum biochemical parameters were found. Our results suggest that the improvement in biochemical parameters are mainly caused by CR, while improvements in anthropometric parameters are further enhanced by TRE.

## 1. Introduction

Obesity is a complex health condition characterized by excessive accumulation of body fat due to a combination of genetic, environmental, and behavioral factors. Obesity increases the risk of several chronic diseases, including type 2 diabetes mellitus, cardiovascular disease, kidney and liver disease, and certain types of cancers [1]. In addition, recent data show that obesity and impaired metabolism are important risk factors for severe coronavirus disease (COVID-19) [2]. It also affects overall quality of life and can lead to psychological and social consequences [3]. The prevalence of obesity is increasing worldwide and represents a major public health challenge [4].

There are several strategies for weight loss and weight loss maintenance that vary in macronutrient composition and degree of caloric restriction (CR). Nevertheless, effective strategies to achieve weight loss and long-term weight loss maintenance have proved to be elusive. Optimal diets and exercise plans for weight loss are a topic of debate among researchers, nutrition experts, and the general public. CR is the most important factor and is traditionally recommended in weight loss or weight-management strategies [5]. In recent years, time-restricted eating (TRE) has gained scientific and public attention as an alternative to conventional weight loss strategies [6].

TRE is an eating pattern in which the daily eating window is limited to a specific period of time, usually between 4 and 12 h, with fasting during the remaining hours. TRE differs from other forms of intermittent fasting in that it requires a consistent time of the daily eating window. It aims to align the body’s natural circadian rhythms with the eating schedule [7].

TRE has been shown to have positive effects in humans; it leads to weight loss, increases insulin sensitivity, lowers blood pressure, and has other positive effects on metabolic health [8]. In humans, however, TRE, when practiced under ad libitum conditions, often leads to an unintended reduction in energy intake, which in turn may lead to weight loss and various cardio-metabolic benefits such as improved insulin sensitivity and lower blood pressure. However, it remains uncertain whether these beneficial effects observed in humans are primarily attributed to the unintentional CR that results from fasting alone, or whether they are due to a combination of both factors [7].

Previous studies have also shown that the effects of TRE may depend on the timing of food intake, but the exact timing has not been well defined. It is not yet clear whether differences occur when food intake is restricted to the early or late part of the day [8]. Another interesting unanswered question is whether the efficacy of a caloric restrictive diet is improved by the implementation of a TRE. Some research questions remain unresolved because of the large heterogeneity of studies. Therefore, the main objective of the present systematic meta-analysis, which considers homogeneous and recent research in the field, is to evaluate the health effects of TRE with CR vs. CR. In addition, we aim to perform subgroup analyses of eTRE, lTRE, and mTRE with CR vs. CR to determine which subgroup of TRE with CR is most effective regarding anthropometric and biochemical parameters. This is indeed a new, unexplored, and very interesting topic for research, and for this reason we would like to highlight where further high quality studies are still needed.

## 2. Materials and Methods

### 2.1. Search Strategy

The search strategy was based on keywords. Medical subject headings (MeSH), title/abstract keywords, and free-text search terms were used. Databases searched included PubMed, Web of Science, Cochrane Library, and EMBASE from 20 April 2023 to 25 May 2023. Moreover, grey literature was searched via Greylit and OpenGrey. Search terms included combinations of »time restricted eating«, or »time restricted feeding«, or »time restricted diet« and »calorie restriction«, or »caloric restriction«, or »energy restriction«, or »energetic restriction« and »weight loss«, or »overweight«, or »obesity«. The detailed search strategy for each database is presented in Appendix A. Search criteria were research papers no older than ten years and randomized trials, available in full text and written in English.

**Figure 1:**
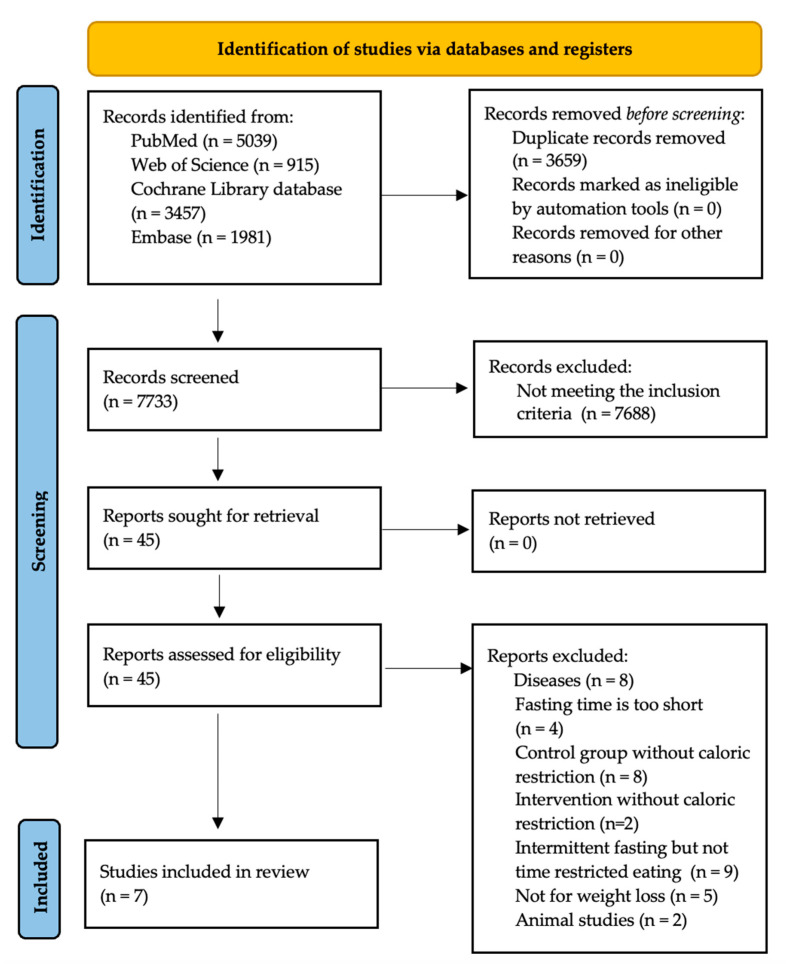
PRISMA flow diagram.

This meta-analysis was registered with the International Prospective Register of Systematic Reviews (PROSPERO) database (registration ID: CRD42023478917). The literature search was conducted according to the recommended protocol of the Preferred Reporting Items for Systematic Reviews and Meta-Analyses (PRISMA) statement and the checklist [9]. All titles and abstracts were screened for eligibility by two independent reviewers (T.Č. and B.H.). Figure 1 shows the detailed search strategy.

From all identified records, 3659 duplicate records were removed. After removing the duplicate records, 7733 titles and abstracts were checked. In addition, 45 full texts were screened using our criteria. A total of 38 studies were excluded because they did not meet our inclusion criteria. Finally, 7 studies were considered for data extraction and analysis (Figure 1). When searching the grey literature, we found no further studies relevant to our analysis.

### 2.2. Study Selection

Eligibility criteria were defined using the PICO framework (Population, Intervention, Comparator, Outcome) [10]. We included studies with the following characteristics: (1) Population: adults aged 18 years or older, with or without metabolic syndrome, and with a BMI > 25 kg/m^2^. (2) Intervention: a daily fasting period with 14–18 h fasting and 6–10 eating windows with CR or a low-carbohydrate diet, two to four months duration. (3) Comparators: a control group in randomized control trials (RCTs) with CR or a low-carbohydrate diet. (4) We divided TRE with CR regimes into: early time-restricted eating (eTRE) with CR, late time-restricted eating (lTRE) with CR, and mTRE with undefined beginning of TRE (eTRE or lTRE) with CR. Specifically, the food intake of eTRE started before 11:00 AM, and the food intake of lTRE started at 11:00 AM or later. (5) Outcomes: data on changes in at least one of the following: body weight (BW), fat-free mass (FFM), fat mass (FM), waist circumference (WC), diastolic blood pressure (DBP), systolic blood pressure (SBP), total cholesterol (TC), low-density lipoprotein cholesterol (LDL), high-density lipoprotein cholesterol (HDL), triglycerides (TG), or fasting glucose (FG). (6) Due to the small number of studies comparing eTRE with lTRE, we also included a study with a low-carbohydrate diet [11] in which energy intake was not monitored, but we assume that spontaneous CR occurred.

We excluded studies with the following characteristics: (1) an intervention or a control group without CR or a low-carbohydrate diet; (2) studies including participants with acute or chronic diseases, such as gastrointestinal diseases, liver/kidney diseases or cancer, type 2 diabetes mellitus, or cardiovascular diseases that affect the outcomes; (3) studies including athletes or participants with intensity physical activity; and (4) animal experiments, meta-analyses, case reports, reviews, conference abstracts, and protocols.

### 2.3. Data Extraction and Collection

Based on the inclusion and exclusion criteria, two authors independently extracted studies into the database (Mendeley Reference Manager). If necessary, a third researcher was consulted. For data that were not available in the articles, we attempted to contact the authors of the articles to obtain information about the missing data. We used Microsoft Excel 2021 to create a table in which all important data from the included studies were entered. The following variables were extracted from each study by these investigators using the same criteria: author’s name, publication year, study group, study duration, outcomes measured, type of intervention, eating window, CR, sample size, and participant characteristics (sex, age, BMI) (Table 1).

### 2.4. Risk of Bias and Certainty of Evidence Assessment

**Figure 2:**
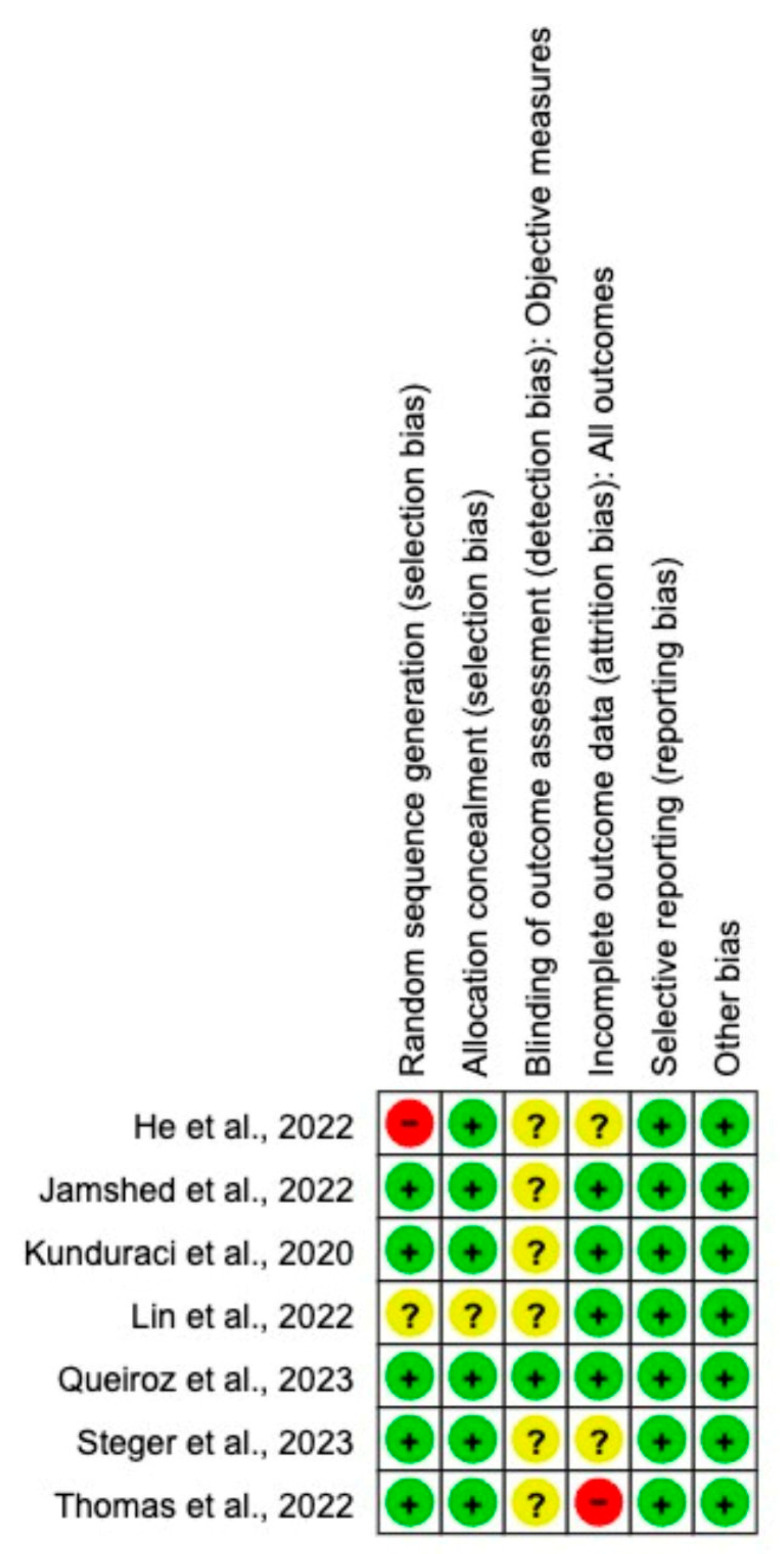
Risk of bias assessment in the RCT s included in the meta-analysis. Criteria for each included RCTs according to Cochrane Risk-of-Bias tool. Abbreviations: −, low risk [7]; ?, unclear risk [13,14,15,16]; +, high risk [11,12] in the PRISMA flow diagram.

The quality assessment of included studies is shown in Figure 2. The Cochrane Collaboration’s tool was used to assess the risk of bias in studies [17]. The risk of bias was assessed by two independent reviewers (T.Č. and B.H.). Bias was assessed as a judgement (high, low, or unclear) for six elements: (1) random sequence generation (selection bias); (2) allocation concealment (selection bias); (3) blinding of outcome assessment (detection bias); (4) incomplete outcome data (attrition bias); (5) selective reporting (reporting bias); and (6) other bias. One study was categorized as having a high risk of incomplete outcome data because the measurements were conducted by participants at home [12], and one study because participants were not randomly assigned to eTRE or lTRE [11]. Another study had three unclear risks of bias, because there was no description of the randomization, allocation, and blinding of outcome process [14] (Figure 2).

We assessed the certainty of the evidence of each outcome using the Grading of Recommendations Assessment, Development, and Evaluation (GRADE) framework [18] (Table 2). The GRADE framework was used was to categorize the quality of evidence of each outcome as very low, low, moderate, and high level by two independent researchers (T.Č. and H.B.). The RCT studies were classified as high quality initially and then downgraded or upgraded depending on predefined criteria, including the risk of bias, consistency, indirectness, and imprecision of results.

### 2.5. Data Analysis and Statistical Methods

Data were analyzed, and forest plots were produced using RewMan version 5.4.1. All results were expressed as mean differences (MDs) with standard deviations (SDs) and/or their 95% confidence intervals (CIs). If the SD of outcomes’ indicators were not available, SDs were calculated using standard errors or confidence intervals for group means based on the approach described in the Cochrane Handbook for Systematic Reviews of intervention [19]. We assumed a correlation of 0.75 from baseline to follow-up measurements to calculate missing values. *I*^2^ was used to represent the heterogeneity of the results (0 to 40% for possibly not significant; 30 to 60% for may indicate moderate heterogeneity; 50 to 90% for may indicate significant heterogeneity; 75 to 100% for significant heterogeneity). Therefore, the random-effect model was [7,11,12,13,14,15,16] used if *I*^2^ > 50%; otherwise, the fixed-effect model was used. *p* < 0.05 was considered statistically significant. We used a random-effect model when the set of studies was heterogeneous and inconsistent (*I*^2^ > 50%), because the random-effect meta-analysis gave the studies proportionally more weight than they would have received in a fixed-effect meta-analysis. A fixed-effect model was used when *I*^2^ < 50%, which meant that studies with low heterogeneity were pooled in the meta-analysis.

## 3. Results

### 3.1. Effects of TRE with CR vs. CR on Changes in Anthropometric Parameters

Seven studies were compared. Two different interventions (eTRE and lTRE) were implemented in two studies.

**Figure 3:**
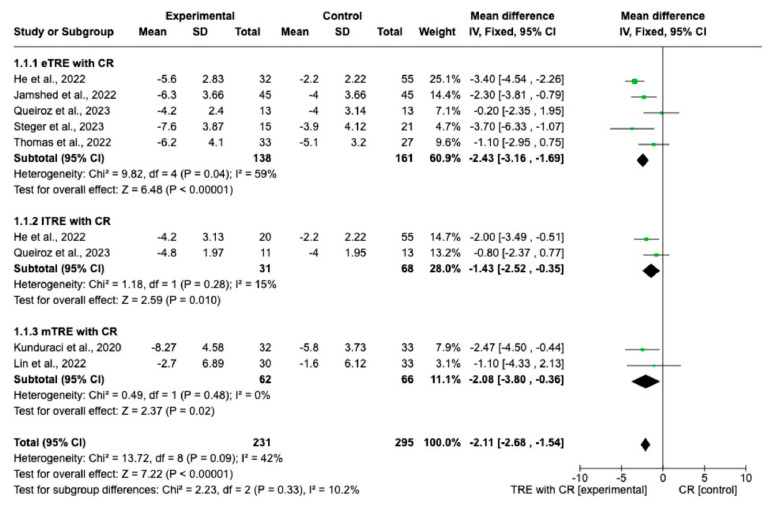
Effect of TRE with CR vs. CR on changes in BW (in kg). Interventions divided into subgroups (eTRE, lTRE, mTRE). MD indicates the mean difference of change from TRE with CR vs. CR. The plotted points are the MDs, and the horizontal error bars represent the 95% confidence intervals. Abbreviations: CR, caloric restriction; eTRE, early time-restricted eating; lTRE, late time-restricted eating; TRE, time-restricted eating; mTRE, undefined beginning of time-restricted eating. The diamond at the base of the plot demonstrates the pooled effect estimates and confidence intervals from all RCTs included in the meta-analysis [7,11,12,13,14,15,16].

**Figure 4:**
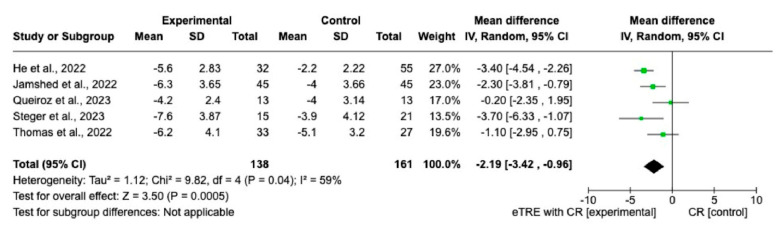
Random effect of TRE with CR vs. CR on changes in BW (in kg). MD indicates the mean difference of change from eTRE with CR vs. CR. The plotted points are the MDs, and the horizontal error bars represent the 95% confidence intervals. Abbreviations: CR, caloric restriction; eTRE, early time-restricted eating. The diamond at the base of the plot demonstrates the pooled effect estimates and confidence intervals from all RCTs included in the meta-analysis [7,11,12,13,16].

Changes in BW were measured in all nine interventions [7,11,12,13,14,15,16] including 231 participants in the TRE group with CR and 227 participants in the control group with CR. The MD of BW change between groups using a fixed-effect model was −2.11 kg (95% CI: −2.68 kg to −1.54 kg, *p* = < 0.00001, *I*^2^ = 42%) (Figure 3), indicating a significantly greater BW loss in the TRE with CR group compared with the CR group. Both, eTRE and lTRE subgroups with CR, compared to the control group with CR resulted in a significantly greater BW loss. The random-effect model (Figure 4) for eTRE [7,11,12,13,16] showed an additional loss of −2.19 kg BW compared to CR (95% CI: −3.42 kg to −0.96 kg; *p* < 0.0005, *I*^2^ = 59%) and the fixed-effect model for lTRE showed an additional loss of −1.43 kg BW compared to CR (95% CI: −2.52 kg to −0.35 kg, *p* = 0.01, *I*^2^ = 15%) [7,11]. In addition, the mTRE subgroup also showed a significant additional BW reduction of −2.08 kg compared to CR (95% CI: −3.80 kg to −0.36 kg, *p* = 0.02, *I*^2^ = 0%) (Figure 3) [14,15].

**Figure 5:**
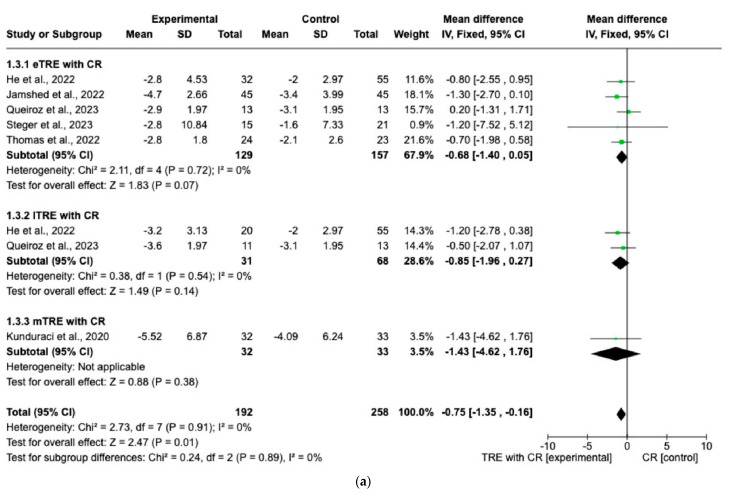

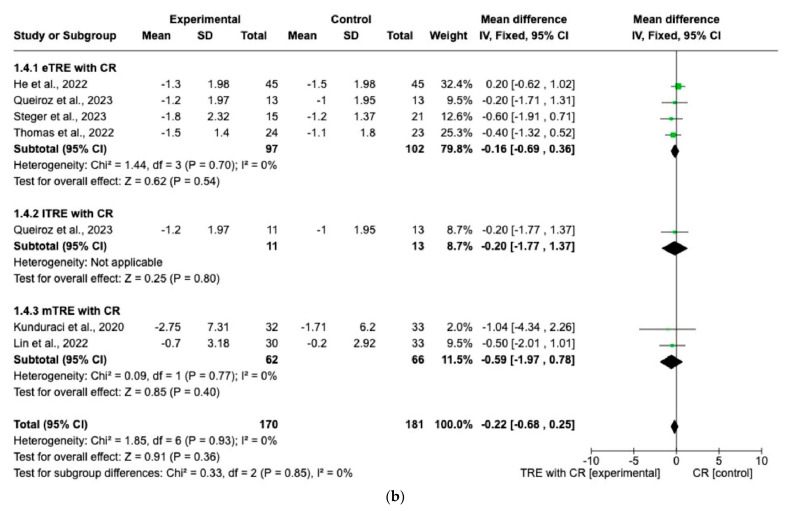
Effect of TRE with CR vs. CR on changes in (**a**) FM (in kg) and (**b**) FFM (in kg). Intervent-ions were divided into subgroups (eTRE, lTRE, mTRE). MD indicates the mean difference of change from TRE with CR vs. CR. The plotted points are the MDs, and the horizontal error bars represent the 95% confidence intervals. Abbreviations: CR, caloric restriction; eTRE, early time-restricted eating; lTRE, late time-restricted eating; TRE, time-restricted eating; mTRE, undefined beginning of time-restricted eating. The diamond at the base of the plot demonstrates the pooled effect estimates and confidence intervals from all RCTs included in the meta-analysis [7,11,12,13,14,15,16].

Changes in FM were evaluated across eight interventions [7,11,12,13,15,16], encompassing 192 participants in the TRE with CR group and 190 in the CR group. Overall, the TRE with CR group exhibited a significantly greater reduction in FM than the control group with CR (MD: −0.75 kg, 95% CI: −1.35 kg to −0.16 kg, *p* = 0.01; *I*^2^ = 0%) (Figure 5a).

However, in both the eTRE and lTRE subgroups, TRE with CR was found to have a small and statistically non-significant effect on FM compared to the control group with CR (MD: −0.68 kg, 95% CI: −1.40 kg to 0.05 kg, *p* = 0.07; *I*^2^ = 0% for eTRE and MD: −0.85 kg, 95% CI: −1.96 kg to 0.27 kg, *p* = 0.14; *I*^2^ = 0% for lTRE). Because there was only one study [15] in the mTRE subgroup, no pooled analysis was possible (Figure 5a). Moreover, seven interventions included FFM as an outcome with 338 individuals (170 in the TRE with CR group and 168 in the control group with CR). There was no significant difference in FFM changes in the TRE with CR group vs. the control group with CR (MD: −0.22 kg, 95% CI: −0.68 kg to 0.25 kg, *p* = 0.36, *I^2^* = 0%) (Figure 5b). The same was shown for the eTRE and mTRE subgroups (MD: −0.16 kg, 95% CI: −0.69 to 0.36 kg, *p* = 0.54, *I*^2^ = 0% for eTRE and MD: −0.59 kg, 95% CI: −1.97 to 0.78 kg, *p* = 0.40, *I*^2^ = 0% for mTRE). Since there was only one study in the lTRE subgroup, no pooled analysis was possible (Figure 5b).

**Figure 6:**
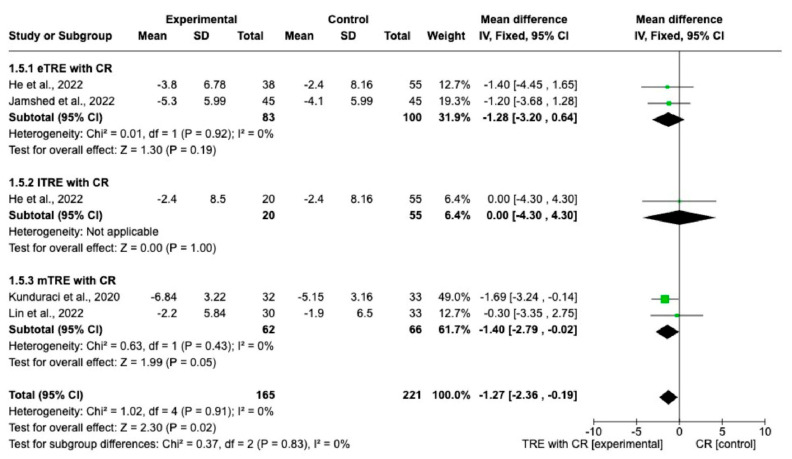
Effect of TRE with CR vs. CR on WC (in cm). Interventions divided in subgroups (eTRE, lTRE, mTRE). MD indicates the mean difference of change from TRE with CR vs. CR. The plotted points are the MDs, and the horizontal error bars represent the 95% confidence intervals. Abbreviations: CR, caloric restriction; eTRE, early time-restricted eating; lTRE, late time-restricted eating; TRE, time-restricted eating; mTRE, undefined beginning of time-restricted eating. The diamond at the base of the plot demonstrates the pooled effect estimates and confidence intervals from all RCTs included in the meta-analysis [11,14,15,16].

Changes in WC were reported in five interventions (165 participants in the TRE with CR group, 166 in control group with CR). The results showed a statistically significantly greater reduction of WC in the TRE group with CR compared with the control group with CR (MD: −1.27 cm, 95% CI: −2.36 cm to −0.19 cm, *p* = 0.02, *I*^2^ = 0%) (Figure 6). In both the eTRE [11,16] and mTRE [14,15] groups, a small and statistically non-significant effect was observed regarding WC compared to the control group with CR (MD: −1.28 cm, 95% CI: −3.20 cm to 0.64 cm, *p* = 0.19, *I*^2^ = 0% for eTRE and MD: −1.40 cm, 95% CI: −2.79 cm to −0.02 cm, *p* = 0.05, *I*^2^ = 0% for mTRE). In the subgroup lTRE [11], pooled analysis was not possible (Figure 6).

**Figure 7:**
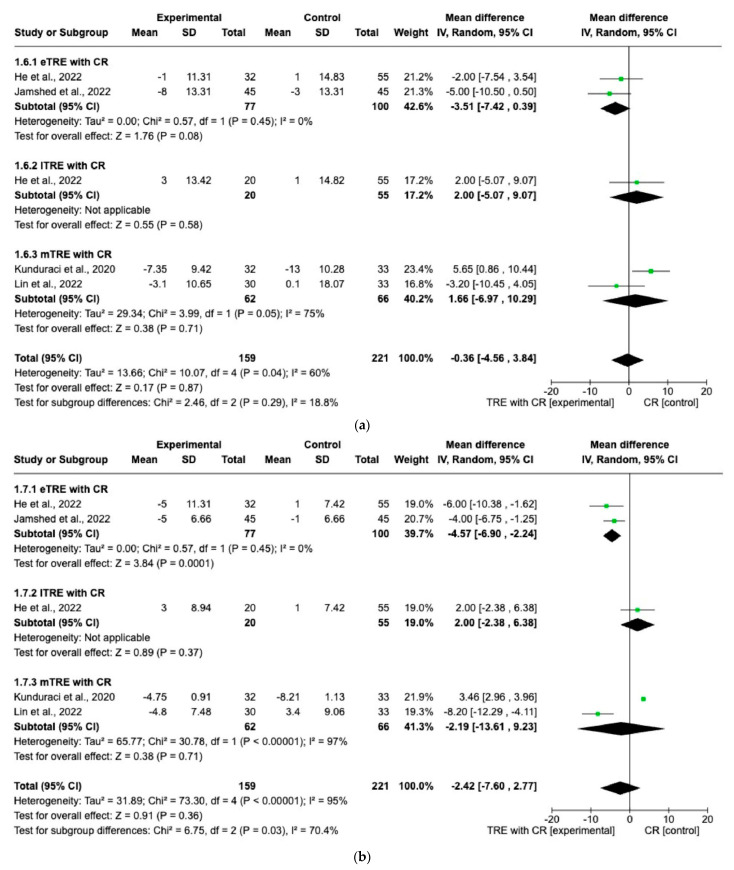
Effect of TRE with CR vs. CR on changes in (**a**) SBP (in mmHg) and (**b**) DBP in (mmHg). Interventions were divided in subgroups (eTRE, lTRE, mTRE). MD indicates the mean difference of change from TRE with CR vs. CR. The plotted points are the MDs, and the horizontal error bars represent the 95% confidence intervals. Abbreviations: CR, caloric restriction; eTRE, early time-res- tricted eating; lTRE, late time-restricted eating; TRE, time-restricted eating; mTRE, undefined beginning of time-restricted eating. The diamond at the base of the plot demonstrates the pooled effect estimates and confidence intervals from all RCTs included in the meta-analysis [11,14,15,16].

Five interventions provided data on SBP and DBP (159 participants in the TRE with CR group, 166 in control group with CR) [11,14,15,16]. The random effect for eTRE with CR subgroup showed a statistically significantly greater decrease in DBP (MD: −4.57 mmHg, 95% CI: −6.90 mmHg to −2.24 mmHg, *p* = 0.0001, *I*^2^ = 0%) (Figure 7a) compared to the control group with only CR [11,16]. On the other hand, there were no significant differences in SBP changes in the eTRE group [11,16] and not even in the mTRE [15,16] subgroup compared with the control group with CR (MD: −3.51 mmHg, 95% CI: −7.42 mmHg to 0.39 mmHg, *p* = 0.08, *I*^2^ = 0% for eTRE and MD: 1.66 mmHg, 95% CI: −6.97 mmHg to 10.29 mmHg, *p* = 0.71, *I*^2^ = 75% for mTRE) (Figure 7a). In the lTRE subgroup [11], no pooled analysis was possible for SBP and DBP (Figure 7a,b).

When performing the random-effect model, no significant difference in changes in SBP and DBP between the TRE group with CR vs. the CR group (MD: −0.36 mmHg, 95% CI: −4.65 mmHg to 3.84 mmHg, *p* = 0.87, *I*^2^ = 60% for SBP and MD: −2.42 mmHg, 95% CI: −7.60 mmHg to 2.77 mmHg, *p* = 0.36, *I*^2^ = 95% for DBP) was shown (Figure 7a,b).

### 3.2. Effects of TRE with CR vs. CR on Changes in Biochemical Parameters

Five interventions, including 131 participants in the TRE group with CR and 124 participants in the control group with CR, evaluated changes in blood levels of FG and lipid profile [7,14,15,16].

**Figure 8:**
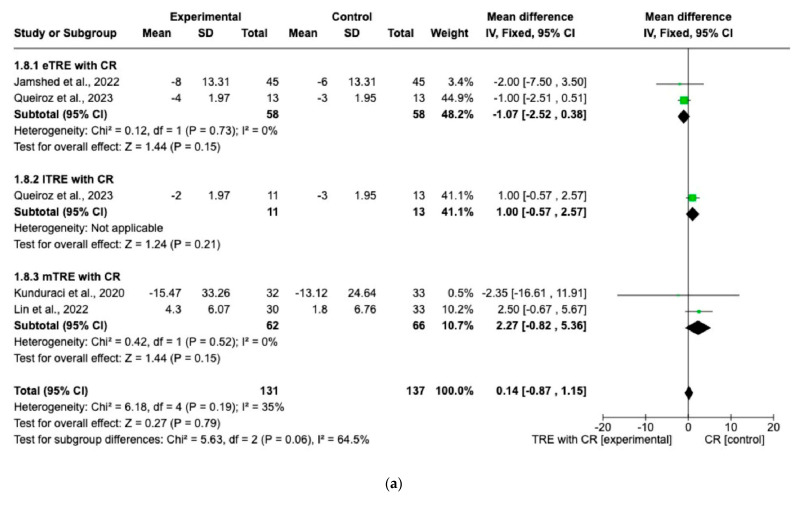

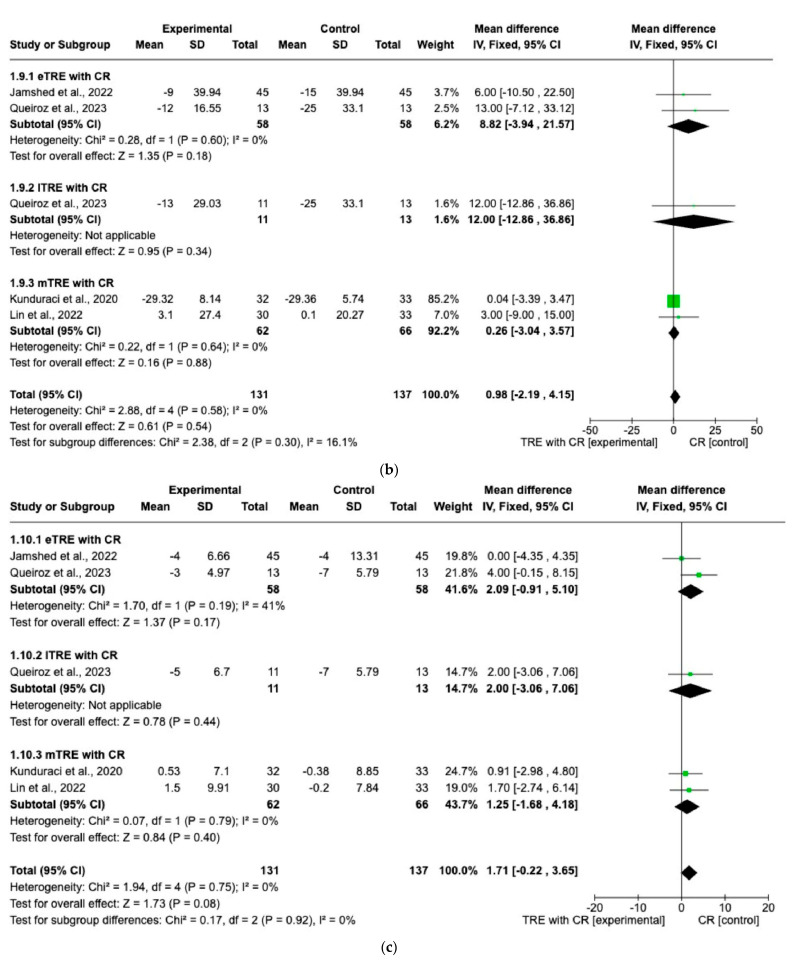

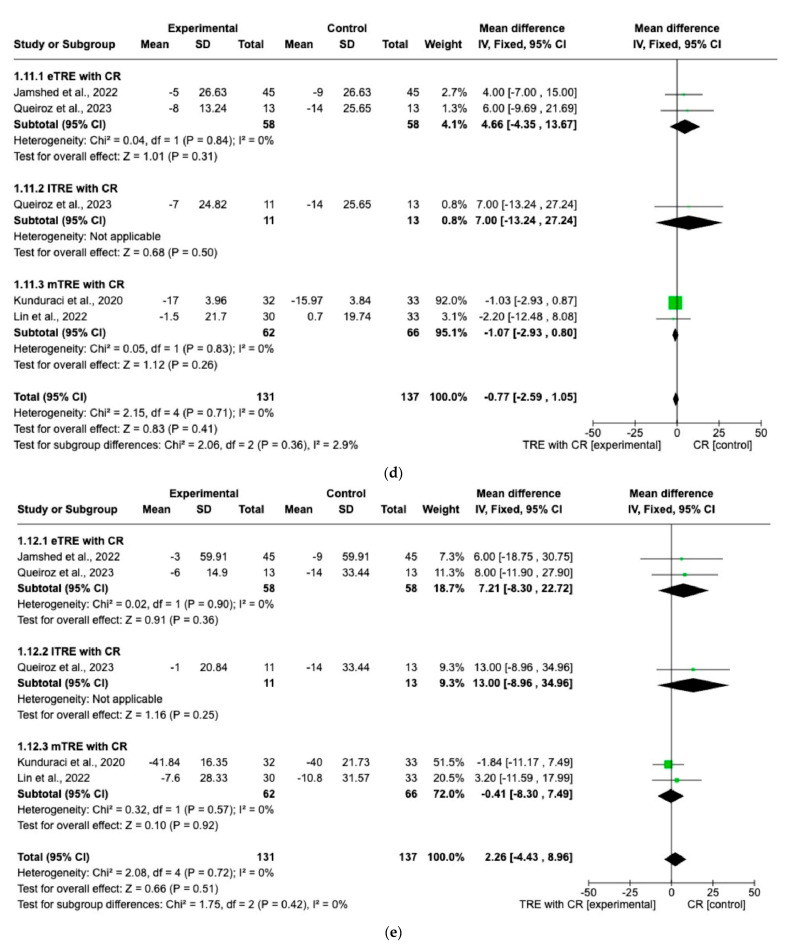
Effects of TRE with CR vs. CR on changes in (**a**) FG (in mg/dL), (**b**) TC (in mg/dL), (**c**) HDL cholesterol (in mg/dL), (**d**) LDL cholesterol (in mg/dL), and (**e**) TG (in mg/dL). Interventions were divided in subgroups (eTRE, lTRE, mTRE). MD indicates the mean difference of change from TRE with CR vs. CR. The plotted points are the MDs, and the horizontal error bars represent the 95% confidence intervals. Abbreviations: CR, caloric restriction; eTRE, early time-restricted eating; lTRE, late time-restricted eating; TRE, time-restricted eating; mTRE, undefined beginning of time-restricted eating. The diamond at the base of the plot demonstrates the pooled effect estimates and confidence intervals from all RCTs included in the meta-analysis [7,14,15,16].

There were no significant differences in FG changes after the interventions between all TRE groups with CR and control groups with CR (MD: 0.14 mg/dL, 95% CI: −0.87 mg/dL to 1.15 mg/dL, *p* = 0.79, *I*^2^ = 35%) (Figure 8a). Similar results were obtained for the lipid profile. Indeed, the fixed-effect model showed no significant difference in TC changes (MD: 0.98 mg/dL, 95% CI: −2.19 mg/dL to 4.15 mg/dL, *p* = 0.54, *I*^2^ = 0%) (Figure 8b), changes in HDL (MD: 1.71 mg/dL, 95% CI: −0.22 mg/dL to 3.65 mg/dL, *p* = 0.08, *I*^2^ = 0%) (Figure 8c), changes in LDL (MD: −0.77 mg/dL, 95% CI: −2.59 mg/dL to 1.05 mg/dL, *p* = 0.41, *I*^2^ = 0%) (Figure 8d), and changes in TG levels (MD: 2.26 mg/dL, 95% CI: −4.43 mg/dL to 8.96 mg/dL, *p* = 0.51, *I*^2^ = 0%) (Figure 8e) between all TRE groups with CR and the control groups with CR.

### 3.3. Certainty of the Evidence

Table 2 presents the GRADE assessment results. Among 11 outcomes analyzed, SBP and DBP were classified as very low quality, and the other nine (BW, BM, FFM, WC, FG, TC, HDL, LDL, TG) were graded as low.

## 4. Discussion

The aim of the present meta-analysis was to evaluate the anthropometric and cardiometabolic effects of TRE with CR vs. CR in overweight and obese adults and to determine which subgroup of TRE is the most effective in combination with CR.

Our meta-analysis included seven studies with nine interventions and 458 participants. The interventions lasted 8 to 14 weeks and included 14 to 16 h of daily fasting with 8 to 10 h eating periods. The control group received CR and in one case a low-carbohydrate diet with no restricted eating window. Outcomes measured included BW, FM, FFM, WC, DBP, SBP, FG, TC, HDL, LDL, and TG. Two studies were categorized as having a high risk of bias, whereas others raised some concerns. The GRADE evaluation rated two of the eleven outcomes from the current study as very low quality, and the remaining nine where classified as low quality.

The main results of our meta-analysis highlight the additive effect of TRE in the presence of CR on anthropometric parameters, especially on BW, FM loss, and WC reduction, compared with CR alone. However, we found no additional impact of TRE in combination with CR on serum biochemical parameters when compared with CR. It is important to note that the analysis of biochemical parameters showed low heterogeneity (*I*^2^ < 35%), indicating consistent results across study participants and variables. In contrast, heterogeneity was higher in the analysis of blood pressure with *I*^2^ values of 60% (SBP) and 95% (DBP), indicating considerable inconsistency or heterogeneity between studies, which may be caused by study design, population characteristics, interventions, or other factors contributing to the observed heterogeneity.

Thus, the main finding from this meta-analysis is that the improvement in biochemical parameters primarily results from CR, while improvements in anthropometric parameters are further enhanced by TRE.

Our findings are consistent with previous studies showing that TRE in combination with CR can significantly affect body composition. Pooled analysis showed a statistically significant and superior effect of the TRE group with CR in terms of BW reduction (average −2.11 kg), FM loss (average −0.75 kg), and WC reduction (average −1.27 cm) compared to the control CR group. Importantly, the aim of dietary strategies is always to reduce BW by maintaining FFM. Indeed, it is important to maintain FFM during weight loss because of its integral role in metabolic rate regulation, preservation of skeletal integrity, and maintenance of functional capacity. In our meta-analysis, the additional reduction in BW in the TRE group with CR did not result in additional reduction in FFM; the mean differences in FFM were not statistically significant between the TRE with CR group and the CR group. The results of a previous meta-analysis are in agreement with our results as they also showed a statistically significant difference regarding BW and FM loss without statistically significant effects on FFM in the TRE with CR vs. CR groups [20]. In individuals with obesity, weight loss is one of the most important strategies to improve health outcomes and prevent or eliminate obesity-related health complications. Data from clinical trials have convincingly shown that a 5% weight loss is considered clinically important [21], and sustained weight loss of 2–5% has shown significant benefits for cardiovascular risk factors [22,23]. However, there is a continuum of clinically important weight loss that varies between individuals and is dependent on comorbidities/complications [21].

Furthermore, the choice of daily eating time appears to have an impact on BW loss and DBP reduction. In our meta-analysis, eTRE with CR and lTRE with CR showed a statistically significant effect on BW vs. CR. In addition, eTRE with CR showed a greater effect on BW than lTRE with CR (eTRE −2.43 kg vs. lTRE −1.43 kg). There was also a statistically significantly greater decrease in DBP in the eTRE with CR subgroup compared to the CR group. A meta-analysis, which also included clinical studies where participants in TRE group ate ad libitum, showed a greater trend of BW reduction in eTRE than lTRE, although the changes in BW were not statistically significant between groups. In the same meta-analysis, the results indicated that eTRE was associated with a higher decrease in DBP compared with non-TRE [24].

One explanation for the anthropometric changes in the TRE groups may be related to the utilization of fatty acids and ketones for energy, because after 6–8 h of fasting, a switch from fat storage to fat utilization occurs [25]. The important consideration related to eTRE vs. lTRE regarding BW loss might be related to the internal circadian clock [26]. Namely, a regulated circadian clock daily regulates the secretion of several hormones and induces a balance between catabolic and anabolic processes. Hormones that are important for regulating metabolism reach their peak secretion in the morning during the active phase. For example, adiponectin, which stimulates fatty acid oxidation and glycolysis and inhibits fat accumulation, is produced between 8 am and 4 pm. Such a mechanism may indicate that the timing for food intake is better in the morning than later in the afternoon [27].

In agreement with previous studies [28,29,30], the main finding of the present meta-analysis is that the improvement in biochemical parameters is mainly caused by CR. TRE with CR had no significant effects on the changes of FG, TC, LDL cholesterol, HDL cholesterol, and TG when compared with CR.

When comparing TRE ad libitum with non-TRE ad libitum, previous studies showed a significant effect of TRE on lowering FG [24,31], whereas the effects of TRE on the lipid profile are less consistent. Some studies have shown no or negative effects on lipid profile [16,20], while others have indicated positive changes in certain lipid profiles [32,33]. A meta-analysis by Liu showed that neither eTRE nor lTRE had a significant effect on lipid profiles in obese or overweight individuals with normal lipid profiles [24]. In the present meta-analysis, serum TC levels decreased more, although not significantly, in the CR group, suggesting a negative effect of TRE in combination with CR on lowering TC levels.

The variability in the effects of TRE on lipid profiles observed in the different studies can be explained by several factors. Participant characteristics are crucial, as some studies focused on overweight or obese individuals with normal lipid profiles, whereas other studies targeted individuals with pre-existing dyslipidemia or other metabolic diseases. Participants’ baseline lipid levels can significantly affect the outcomes [24,32]. Another factor is the differences in TRE interventions that determine the duration of fasting, timing of meals, and overall CR [34]. These differences in the study design can lead to different effects on fat metabolism. For example, the duration of fasting may affect the body’s response to fat metabolism differently. Individual differences in genetic predisposition and metabolic response also influence outcomes [35]. Furthermore, it is also important to consider the limitations of the studies themselves [20,36,37]. We acknowledge that the present study also has some limitations. The main limitation of the present meta-analysis is the inclusion of a study comparing TRE with a low-carbohydrate diet compared with a low-carbohydrate diet, in which we assumed that spontaneous caloric restriction occurred. However, our results did not differ depending on whether the study was included or not. In addition, the study was categorized into eTRE and lTRE, which facilitated the comparison of subgroups. And finally, the quality of the study results was categorized as low or very low according to the GRADE tool. This was primarily attributed to the risk of bias caused mainly by the nature of the behavioral intervention, as it was not possible to blind participants due to the different time frames of fasting and eating. Secondly, it can be attributed to inconsistency, due to the high heterogeneity of some results (e.g., SBP and DBP), and finally it can be attributed to variability in energy restriction protocols. However, the main strength of our meta-analysis compared to other meta-analyses on this topic is that we used only homogeneous studies that included asymptomatic individuals who were overweight or obese with or without components of metabolic syndrome but without chronic diseases and in which fasting time ranged from 14–16 h and study duration was 2–4 months. This approach allowed us to minimize the large heterogeneity of the results, knowing that, for example, the response of individuals with diabetes or some other disorders may be different [20,36,38,39]. In addition, there is also a major problem in interpreting the results more accurately due to the different lengths of the time windows for eating and fasting (8:16–12:12) and, consequently, the small difference between the intervention group and the control group (2 h) [37]. Moreover, the length of studies in other meta-analyses varied from 45 days to 12 months [20,37]. Therefore, longitudinal studies may introduce bias into the results due to non-adherence to the time window and higher attrition. And finally, we would also like to point out that we performed subgroup analyses for eTRE, mTRE, and lTRE. These three subgroups were compared in terms of all anthropometric and biochemical parameters, which has been deficient in other meta-analyses [20,36,37].

## 5. Conclusions

Overall, based on these meta-analysis results, it appears that TRE with CR significantly improves some anthropometric outcomes compared with CR, especially BW, FM, and WC. Moreover, within the eTRE subgroup with CR statistically significant improvements were also shown for DBP. On the other hand, TRE with CR did not lead to significant improvements or differences in FFM, SBP, FG, TC, HDL and LDL cholesterol, and TC compared to the CR group. It is important to consider the limitations of the included studies, such as sample size, duration, and possible differences in study design, which could affect the results presented.

As our results suggest that the improvement in biochemical parameters is mainly caused by CR, while improvements in anthropometric parameters are further enhanced by TRE, TRE with CR could be recommended for individuals with BMI > 25 kg/m^2^ and for those who want to lose BW and FM in a short period of time.

However, further studies are needed to investigate the long-term effects of TRE with CR on anthropometric and metabolic parameters to better understand the potential benefits or limitations of this dietary intervention.

## Figures and Tables

**Table 1:** Baseline characteristics of the included studies.

Study	Country	Study Group (Population)	Duration	Outcomes	Type of Intervention	Eating Window	Caloric Restriction (CR)	Sample Size n (m/f)	Age (Years)	BMI (kg/m^2^)
Queiroz et al. (2023) [7]	Brazil	Overweight and obesity	8 weeks	Changes in BW, FFM, FM, HDL, LDL, TG, TC, FG	eTRE	(8:16) 8:00–16:00	−25% EI	13 (2/11)	33 ± 6	30.0 ± 8.0
					lTRE	(8:16) 12:00–20:00	−25% EI	11 (2/9)	30 ± 7	30.0 ± 5.0
					Non-TRE	8:00–20:00	−25% EI	13 (2/11)	26 ± 4	30.0 ± 1.0
Thomas et al. (2022) [12]	Colorado, USA	Overweight and obesity	12 weeks	Changes in BW, FFM, FM	eTRE	(10:14) Starting within 3 h after waking up	−35% EI	41 (7/34)	38 ± 8	34.6 ± 5.8
					Non-TRE	Unrestricted eating time	−35% EI	40 (5/35)	38 ± 8	33.7 ± 5.6
Steger et al. (2023) [13]	United Kingdom	Obesity	14 weeks	Changes in BW, FFM, FM	eTRE	(8:16) 7:00–15:00	−500 kcal/day	15 (4/11)	46 ± 11	38.5 ± 7.1
					Non-TRE	≥12 h/day	−500 kcal/day	21 (6/15)	42 ± 12	38.3 ± 6.0
Lin et al. (2022) [14]	Taiwan	Normal and overweight women	8 weeks	Changes in BW, FFM, WC, DBP, SBP, HDL, LDL, TG, TC, FG	mTRE	(8:16) 10:00–18:00 or 12:00–20:00	−200 kcal/day	30 (0/30)	50 ± 8	25.9 ± 3.7
					Non-TRE	Unrestricted eating time	−200 kcal/day	33 (0/33)	54 ± 8	25.7 ± 3.8
He et al. (2022) [11]	China	Obesity with metabolic syndrome	12 weeks	Changes in BW, FM, WC, DBP, SBP	eTRE	(8:16) 8:00–16:00	Carbohydrate restriction to < 26% of EI; CR undefined	32 (22/10)	41 ± 9	29.1 ± 3.4
					lTRE	(8:16) 12:00–20:00	Carbohydrate restriction to < 26% of EI; CR undefined	20 (15/5)	37 ± 8	28.8 ± 2.7
					Non-TRE	Unrestricted eating time	Carbohydrate restriction to < 26% of EI; CR undefined	55 (30/25)	41 ± 1	29.3 ± 3.7
Kunduraci et al. (2020) [15]	Turkey	Obesity with metabolic syndrome	12 weeks	Changes in BW, FFM, FM, WC, DBP, SBP, HDL, LDL, TG, TC, FG	mTRE	(8:16) 8:00–16:00 or 9:00–17:00 or 10:00–18:00 or 11:00–19:00	−25% EI	32 (16/16)	47 ± 12	36.6 ± 5.3
					Non-TRE	Unrestricted eating time	−25% EI	33 (15/18)	49 ± 12	32.9 ± 4.1
Jamshed et al. (2022) [16]	Alabama, USA	Obesity	12 weeks	Changes in BW, FFM, FM, WC, DBP, SBP, HDL, LDL, TG, TC, FG	eTRE	(8:16) 7:00–15:00	−500 kcal/day	45 (10/35)	43 ± 10	40.1 ± 6.6
					Non-TRE	≥ 12 h/day	−500 kcal/day	45 (8/37)	43 ± 11	39.2 ± 6.8

Data are presented as mean ± SD. Abbreviations: BW, body weight; CR, caloric restricition; DBP, diastolic blood pressure; FG, fasting glucose; f, female; HDL, high-density lipoprotein cholesterol; EI, energy intake; eTRE, early time-restricted eating; FFM, fat-free mass; FM, fat mass; lTRE, late time-restricted eating; LDL, low-density lipoprotein cholesterol; m, male; mTRE, undifined beginning of time-restricted eating; non-TRE, without time-restricted eating; SBP, systolic blood pressure; TC, total cholesterol; TG, total triglycerides; USA, United States of America; WC, waist circumference.

**Table 2:** Certainty of the evidence.

Certainty Assessment							No. of Patients		Effect		Certainty
No. of Studies	Study Design	Risk of Bias	Inconsistency	Indirectness	Imprecision	Other Considerations	TRE with CR	CR	Relative (95% CI)	Absolute (95% CI)	
BW											
9	randomized trials	serious	not serious	not serious	serious	none	231	295	-	MD **2.11 lower** (2.68 lower to 1.54 lower)	Low
BM											
8	randomized trials	serious	not serious	not serious	serious	none	192	258	-	MD **0.75 lower** (1.35 lower to 0.16 lower)	Low
FFM											
7	randomized trials	serious	not serious	not serious	serious	none	170	181	-	MD **0.22 lower** (0.68 lower to 0.25 higher)	Low
WC											
5	randomized trials	serious	not serious	not serious	serious	none	165	221	-	MD **1.27 lower** (2.36 lower to 0.19 lower)	Low
SBP											
5	randomized trials	serious	serious	not serious	serious	none	159	221	-	MD **0.36 lower** (4.56 lower to 3.84 higher)	Very low
DBP											
5	randomized trials	serious	very serious	not serious	serious	none	159	221	-	MD **2.42 lower** (7.6 lower to 2.77 higher)	Very low
FG											
4	randomized trials	serious	not serious	not serious	serious	none	131	137	-	MD **0.14 higher** (0.87 lower to 1.15 higher)	Low
TC											
5	randomized trials	serious	not serious	not serious	serious	none	131	137	-	MD **0.98 higher** (2.19 lower to 4.15 higher)	Low
HDL											
5	randomized trials	serious	not serious	not serious	serious	none	131	137	-	MD **1.71 higher** (0.22 lower to 3.65 higher)	Low
LDL											
5	randomized trials	serious	not serious	not serious	serious	none	131	137	-	MD **0.77 higher** (2.59 lower to 1.05 higher)	Low
TG											
5	randomized trials	serious	not serious	not serious	serious	none	131	137	-	MD **2.26 higher** (4.43 lower to 8.96 higher)	Low

Abbreviations: BW, body weight; CR, caloric restricition; DBP, diastolic blood pressure; FG, fasting glucose; HDL, high-density lipoprotein cholesterol; FFM, fat-free mass; FM, fat mass; LDL, low-density lipoprotein cholesterol; MD, mean difference; SBP, systolic blood pressure; TC, total cholesterol; TG, total triglycerides; TRE, time-restricted eating; WC, waist circumference.

## Data Availability

Data is contained within the article.

## Appendix A. Search Strategy for Each Database

1. Pubmed

(((((((((time restricted eating[Title/Abstract]) OR (time restricted feeding[Title/Abstract])) OR (time restricted diet[Title/Abstract])) AND (calorie restriction[Title/Abstract])) OR (energy restriction[Title/Abstract])) OR (caloric restriction[Title/Abstract])) AND (overweight[MeSH Terms])) OR (obesity[MeSH Terms])) OR (metabolic syndrome[MeSH Terms])) OR (weight loss[MeSH Terms])

2. Web of Science

Results for TIME RESTRICTED EATING (Title) OR TIME RESTRICTED FEEDING (Title) OR TIME RESTRICTED DIET (Title) AND CALORIC RESTRICTION (Title) OR CALORIE RESTRICTION (Title) OR ENERGY RESTRICTION (Title) and Open Access and 2023 or 2022 or 2021 or 2020 or 2019 or 2010 or 2011 or 2012 or 2013 or 2014 or 2015 or 2016 or 2017 or 2018 (Publication Years) and Article (Domcument Types).

3. Cochrane library

#1 (Time restricted feeding): ti, ab, kw
#2 (Time restricted eating): ti, ab, kw
#3 (Time restricted diet); ti, ab, kw
#4 #1 or #2 or #3
#5 (Calorie restriction): ti, ab, kw
#6 (Energy restriction): ti, ab, kw
#7 (Caloric restriction): ti, ab, kw
#8 #5 or #6 or #7
MeSH descriptor:
#9 (overweight)
#10 (obesity)
#11 (obese)
#12 (metabolic syndrome)
#13 (weight loss)
#14 (weight reduction)
#15 #9 or #10 or #11 or #12 or #13 or #14
#16 #4 and #8 and #15

4. Embase

Time restricted eating OR time restricted feeding OR time restricted eating OR time restricted diet OR time restricted feeding AND calorie restriction OR caloric restriction OR energy restriction AND weight loss OR overweight OR obesity OR metabolic syndrome.

## Appendix B

**Table A1:** 2020 Checklist.

Section and Topic	Item #	Checklist Item	Location Where Item Is Reported
TITLE			
Title	1	Identify the report as a systematic review.	Title page
ABSTRACT			
Abstract	2	See the PRISMA 2020 for abstract checklist.	Title page
INTRODUCTION			
Rationale	3	Describe the rationale for the review in the context of existing knowledge.	Section 1
Objectives	4	Provide an explicit statement of the objective(s) or question(s) the review addresses.	Section 1
METHODS			
Eligibility criteria	5	Specify the inclusion and exclusion criteria for the review and how studies were grouped for the syntheses.	Section 2.2
Information sources	6	Specify all databases, registers, websites, organisations, reference lists, and other sources searched or consulted to identify studies. Specify the date when each source was last searched or consulted.	Section 2.1
Search strategy	7	Present the full search strategies for all databases, registers, and websites, including any filters and limits used.	Section 2.1 and Appendix A
Selection process	8	Specify the methods used to decide whether a study met the inclusion criteria of the review, including how many reviewers screened each record and each report retrieved; whether they worked independently; and if applicable, details of automation tools used in the process.	Section 2.1
Data collection process	9	Specify the methods used to collect data from reports, including how many reviewers collected data from each report; whether they worked independently; any processes for obtaining or confirming data from study investigators; and if applicable, details of automation tools used in the process.	Section 2.3
Data items	10 a	List and define all outcomes for which data were sought. Specify whether all results that were compatible with each outcome domain in each study were sought (e.g., for all measures, time points, analyses), and if not, the methods used to decide which results to collect.	Section 2.2
	10 b	List and define all other variables for which data were sought (e.g., participant and intervention characteristics, funding sources). Describe any assumptions made about any missing or unclear information.	Section 2.1 and Section 2.2
Study risk of bias assessment	11	Specify the methods used to assess risk of bias in the included studies, including details of the tool(s) used; how many reviewers assessed each study and whether they worked independently; and if applicable, details of automation tools used in the process.	Section 2.4
Effect measures	12	Specify for each outcome the effect measure(s) (e.g., risk ratio, mean difference) used in the synthesis or presentation of results.	Section 2.4
Synthesis methods	13 a	Describe the processes used to decide which studies were eligible for each synthesis (e.g., tabulating the study intervention characteristics and comparing against the planned groups for each synthesis (item #5)).	Section 2.2 and Figure 1
	13 b	Describe any methods required to prepare the data for presentation or synthesis, such as handling of missing summary statistics, or data conversions.	Section 2.4
	13 c	Describe any methods used to tabulate or visually display results of individual studies and syntheses.	Section 2.4
	13 d	Describe any methods used to synthesize results and provide a rationale for the choice(s). If meta-analysis was performed, describe the model(s) and method(s) to identify the presence and extent of statistical heterogeneity, as well as software package(s) used.	Section 2.4 and Section 2.5
	13 e	Describe any methods used to explore possible causes of heterogeneity among study results (e.g., subgroup analysis, meta-regression).	Section 2.5
	13 f	Describe any sensitivity analyses conducted to assess robustness of the synthesized results.	Section 2.4
Reporting bias assessment	14	Describe any methods used to assess risk of bias due to missing results in a synthesis (arising from reporting biases).	Figure 2
Certainty assessment	15	Describe any methods used to assess certainty (or confidence) in the body of evidence for an outcome.	Section 2.4 and Table 2
RESULTS			
Study selection	16 a	Describe the results of the search and selection process, from the number of records identified in the search to the number of studies included in the review, ideally using a flow diagram.	Figure 1
	16 b	Cite studies that might appear to meet the inclusion criteria but were excluded, and explain why they were excluded.	Figure 1
Study characteristics	17	Cite each included study and present its characteristics.	Table 1
Risk of bias in studies	18	Present assessments of risk of bias for each included study.	Figure 2
Results of individual studies	19	For all outcomes, present, for each study: (a) summary statistics for each group (where appropriate) and (b) an effect estimate and its precision (e.g., confidence/credible interval), ideally using structured tables or plots.	Figure 3, Figure 4, Figure 5, Figure 6, Figure 7 and Figure 8
Results of syntheses	20 a	For each synthesis, briefly summarize the characteristics and risk of bias among contributing studies.	Table 1
	20 b	Present results of all statistical syntheses conducted. If meta-analysis was done, present for each the summary estimate and its precision (e.g., confidence/credible interval) and measures of statistical heterogeneity. If comparing groups, describe the direction of the effect.	Section 3
	20 c	Present results of all investigations of possible causes of heterogeneity among study results.	Section 3
	20 d	Present results of all sensitivity analyses conducted to assess the robustness of the synthesized results.	Section 3
Reporting biases	21	Present assessments of risk of bias due to missing results (arising from reporting biases) for each synthesis assessed.	Figure 2
Certainty of evidence	22	Present assessments of certainty (or confidence) in the body of evidence for each outcome assessed.	Table 2
DISCUSSION			
Discussion	23 a	Provide a general interpretation of the results in the context of other evidence.	Section 4
	23 b	Discuss any limitations of the evidence included in the review.	Section 4
	23 c	Discuss any limitations of the review processes used.	Section 4
	23 d	Discuss implications of the results for practice, policy, and future research.	Section 4
OTHER INFORMATION			
Registration and protocol	24 a	Provide registration information for the review, including register name and registration number, or state that the review was not registered.	Section 2.1
	24 b	Indicate where the review protocol can be accessed, or state that a protocol was not prepared.	N
	24 c	Describe and explain any amendments to information provided at registration or in the protocol.	N
Support	25	Describe sources of financial or non-financial support for the review, as well as the role of the funders or sponsors in the review.	Funding
Competing interests	26	Declare any competing interests of the review authors.	Conflicts of Interest
Availability of data, code, and other materials	27	Report which of the following are publicly available and where they can be found: template data collection forms; data extracted from included studies; data used for all analyses; analytic code; and any other materials used in the review.	N
